# Supplementary material for: Patterns of Intron Gain and Loss in Fungi
Source: PLoS Biol. 2004 Nov 30;2(12):e422. doi: 10.1371/journal.pbio.0020422 (PMC532390; doi:10.1371/journal.pbio.0020422)
Supplement: Table S1 — Also available at http://genes.mit.edu/NielsenEtAl/. (4.3 MB ZIP). [file pbio.0020422.st001.zip › NielsenEtAl/html/367.html]

AN0091.1.NCU06266.1.MG05254.1.FG02567.1


```
 CLUSTAL W (1.82) Multiple Sequence Alignments - Introns Inserted


Sequence 1: MG05254.1	510 aa
Sequence 2: FG02567.1	493 aa
Sequence 3: NCU06266.1	531 aa
Sequence 4: AN0091.1	501 aa
Alignment Length: 564 aa
Number Identitical Residues: 150 aa
Alignment Score (without introns) 8099


MG05254.1 	---MGILAQKS-KFKIAKPTIRVEKVAVEVPQARAKPSRP---------------LGGGG
NCU06266.1	---MSIFNQKS-KFKIKT-EIRTVRETVERPKLKASTSSSSIPTTGSSQHSNVRIAGAGS
FG02567.1 	---MRLFGGKNNKFKVDPPKIRIEKVVVDRPAQKPKPKPN---------------PALSG
AN0091.1  	MGGFDYLQKGGTGFTLQVKKPQIRRVVQTRP---AAPSPS--------------------
          	 ..:  :   .. *.:     :  : .   *   . ..                      

MG05254.1 	ADRASSGSPRPQQPQPPRVRASAS--------PLPFASSSRASSSLTSSGAQ-QYQRKRK
NCU06266.1	SLSRASSTPRLNGTSHTTSTSSSARASLTKVTTIPTARRIQSASPIPSINGDGRSSRKRR
FG02567.1 	SASRSSSSHHPS-PKPLARQASHS--------PYP-----------SSCDEK-RLERKRK
AN0091.1  	-ANKATPRTVPSGPQKKTPETASR----------------------SVTGERGFSPSKRR
          	    ::     . ..     ::                        .  .  .    **:

MG05254.1 	ASSAGPAAAPRRSPANSDRVTWDQESDSEDDNAWEDTLDDRKRQRTTDSERAEDPNRRLV
NCU06266.1	AIKAS-----SRSPI-SPHFSEDSDSDSNGDENWEDNLDARKRRKRVHDERPVDVNRKLR
FG02567.1 	APSVS-----RKSPA-SDRIEFDKDSDGEDD-GWM-TLDTKRQRKGTEDSGFVDPNRKLR
AN0091.1  	LTPLRN----RKRPTPEQRLSSDDDDDGSDT---DTSLELRKRARTGESAEP-DYGRRLR
          	           : *  . :.  *.:.*...      .*: ::: :  ..    * .*:* 

MG05254.1 	SPALTD-PPPDGRD----LRLIHAADVASLKL--KCTPSLGAPASDVRVALQYPGLRQRE
NCU06266.1	HPKIWKGEEADQVERIPGPAIIHAAKVASLEH--KCKPALGLKNEEVAVRLQYPGARYPE
FG02567.1 	SVRAFE-ERMDSR------KFIHAVDVASLEH--KCVPVMGAQKDEVAIRLQYPSLQPRE
AN0091.1  	SLKAFSGDETRSLP------IVHASEITSVQKPGKFKPAFENMNQTSEIFLQYPSATPKE
          	     ..             ::** .::*:: ..*  * :    .   : ****.    *

MG05254.1 	R2YELVSGKD--MIDAVEEIKHVINCVVDTYLTENEARPFTDANNGLIRQLERSS--NLK
NCU06266.1	R2YELVEGKD--KIDAVKDIEKVVQLAASTYLTDEEGKIFLDHQTGILRKLVKSK--NRS
FG02567.1 	K2YELVWGKD--KIDAVEASIKVVRHVAETYLTEEEAEPFTNPNGGIIRRLEKASNRNIQ
AN0091.1  	R2YEAVVPRDDDEFKPLDDIVQVIETVTQAYIPEDELDEFNNESTGIKRRLRRAL--ARG
          	: ** *  :*.. :..:.   :*:. ...:*:.::*   * : . *: *:* ::      

MG05254.1 	DYKAFRAALDKYNLRLRELQESGAIARNLAKRHLLPFNLVQLILQQVYDRTVAPRVELLA
NCU06266.1	DGPGFKAALREYNDEFLKLMRKGVIVRNLDKMHGIPRELVDFILDQVYDRTVAPRVELLA
FG02567.1 	DLMGFKAALREYNEKLRALVDDGVIAKNLDKMHELPQHLVAFILDQIYDRTVALKVELLS
AN0091.1  	SEREFRESVKDYNVAIERLRRSGSIAKKLDATYRLSLPHVERILTQIYSRTVSPRVDSLR
          	.   *: :: .**  :  *  .* *.::*   : :.   *  ** *:*.***: :*: * 

MG05254.1 	KYENGTDNVYGELLHPFISEILVQRLKMRSDQVFVDLGSGVGNVVLQAALDIGCESWGCE
NCU06266.1	KYQNGSDNVYGELNHPFISDIL-ERTNLTSDKVFVDLGSGVGNVTLQAALEIGCESWGCE
FG02567.1 	KYENGTDYVYGELLHPFISKVLVEQTRMTSGQVFVDLGSGVGNVVLQAALEIGCESWGCE
AN0091.1  	QYENGTDNVYGELLPRFISTIF-KETGLKSNHVFVDLGSGVGNVVLQAALEIGCESWGCE
          	:*:**:* *****   *** :: :.  : *.:************.*****:*********

MG05254.1 	MMENACNLADAQKKEFTARCRLWGIAPGKVRLERGDFRTNERTLSALKRADVVLVNNQAF
NCU06266.1	MMENACNLAEAQYDEFLARCRLWGVKPGKVRLERGDFRKNEKILEVLKRADVVLVNNQAF
FG02567.1 	MMENACNLAEEQKKEFDARCMLWGVRPGKVHLERGDFRKNAPIHEALKRADVVLVNNKAF
AN0091.1  	MMQNACDLAELQQAEFKARCRLWGIAPGKTHLVRGDFLKEQSIIDVLKRADVVLINNQAF
          	**:***:**: *  ** *** ***: ***.:* **** .:    ..********:**:**

MG05254.1 	TAQLNDDLVRIFLDLRKGCKIVSLKSFVHEQKN--AANDVG-SSILDVERLSYPEGYVSW
NCU06266.1	TSQLNDNLVSMFLDLKIGCKIISLKTFVHDNKN--AENDVA-TSILDVEHLTYPEGYVSW
FG02567.1 	TSQLNDDLVRMFLDLKSGCKVVSLKSFVAEKSNNHNINDVG-STILEVEECIYPEGYVSW
AN0091.1  	TPQLNNELINHFLDMKEGCQIVSLKSFVPVGHKIQSRNLNSPINLLTVKQRQYWSNSVSW
          	*.***::*:  ***:: **:::***:**    :    *  .. .:* *:.  * .. ***

MG05254.1 	TGAGGQFCISTRK------------
NCU06266.1	TGASGTYCISTRR------------
FG02567.1 	TNAGGSYFISTRK------------
AN0091.1  	TDVGGSYFIATKDSSRLKAFSESLA
          	*...* : *:*: ::  .: :.: :
```
